# A review of venous thromboembolism risk assessment models for different patient populations: What we know and don’t!

**DOI:** 10.1097/MD.0000000000032398

**Published:** 2023-01-13

**Authors:** Y Mehta, A Bhave

**Affiliations:** a Medanta Institute of Critical Care and Anaesthesiology, Medanta—The Medicity, Gurgaon, Haryana, India; b Lilavati Hospital and Research Centre, Mumbai, Maharashtra, India.

**Keywords:** Caprini, India, Padua, prophylaxis, risk assessment model (RAM), risk factors, venous thromboembolism (VTE)

## Abstract

Venous thromboembolism (VTE) is a common cause of morbidity and mortality in hospitalized patients. Globally, it is also the third leading vascular disease, after myocardial infarction and stroke. The incidence of VTE is reportedly higher in Western countries than in Asian countries. However, recent reports suggest an increasing incidence of VTE in Asian countries, including India. Since VTE is largely a preventable disease, early identification of risk factors can lead to disease prevention or the adoption of appropriate prophylactic measures. To this end, several VTE risk assessment models (RAMs) have been developed and validated for different populations who are at risk of developing VTE, such as hospitalized patients with medical illness/surgical indication, patients with cancer, and pregnant women. Evidence indicates that the systematic use of RAMs improves prophylaxis rates and lowers the burden of VTE. Given the increasing burden of VTE in the Indian population and poor prophylaxis rates, the implementation of systematic RAMs in routine clinical practice might ameliorate the disease burden in the country. We have assessed the evidence-based utilities of available RAMs and have delineated the most common and suitable RAMs for different populations including coronavirus disease 2019 affected patients. This review depicts the current status of implementation and validation of RAMs in the Indian scenario. It also highlights the need for additional validation studies, improved awareness, and implementation of RAMs in clinical practice for lowering the burden of VTE.

## 1. Introduction

Venous thromboembolism (VTE), a major contributor to the global disease burden, is the third leading vascular disease, after acute myocardial infarction and stroke, and is associated with significant morbidity and mortality.^[1,2]^ As evident from the reports from different countries, the overall incidence of VTE is 1 to 2 per 1000 people per year.^[1–4]^ The incidence is higher in the elderly.^[5]^ Although the incidence of VTE in Asian countries is significant and increasing with time, it is assumed to be lower as compared to the Western population.^[6–8]^

### 1.1. VTE in India: A growing burden

The Indian subset data of the ENDORSE multinational study found 53.6% of all in-hospital patients to be at risk for VTE.^[9]^ A 5-year multicenter retrospective registry (ARRIVE), involving 3 tertiary care hospitals and 549 medical records of both in and outpatients with a confirmed diagnosis of VTE, estimated that 23%, 64%, and 13% of patients had acute deep-vein thrombosis (DVT) with pulmonary embolism (PE), acute DVT without PE, and PE alone, respectively.^[10]^ The DETECT-DVT registry reported a 3.2% incidence of DVT in Indian acute sepsis patients not receiving thromboprophylaxis.^[11]^ In the RAVS^[12]^ study which was a single center retrospective study, the reported incidence rate of VTE was 14.2 per 100,000 admissions. The christian medical college Vellore study however reported an incidence of 17.4 VTE patients per 10,000 admissions.^[13]^ The limited existing studies on the incidence of VTE in India indicate that in contrast to the previous perception, VTE is no more a rare phenomenon in the country.^[12]^

### 1.2. Risk factors for VTE

VTE is a multifactorial disease resulting from an interplay of acquired and hereditary/genetic risk factors (Table 1).^[14]^ Genetic risk factors include loss-of-function mutations, such as deficiencies in protein S, protein C, or antithrombin III, and gain-of-function mutations, such as factor V Leiden, prothrombin mutation G20210A and JAK2V617F mutation.^[14–17]^ However, prothrombin mutation G20210A is not common in India.^[15,18]^

**Table 1:** Risk factors for venous thromboembolism.^[1,5,10,12–14,19,20]^

Acquired risk factors		Hereditary/Genetic risk factors	
Transient	Persistent	Loss-of-function mutations	Gain-of-function mutations
Surgery (general/orthopedic) and hospitalization	Active malignancy	Deficiency of antithrombin III	Factor V Leiden
Pregnancy and the postpartum period	Overweight and obesity	Deficiency of protein C	Prothrombin
Infections	Chronic inflammatory diseases*	Deficiency of protein S	Mutation G20210A
Trauma	Increasing age	PNH	Elevation of factor VIII
Hormone replacement therapy (oral)	Height		JAK2 V617F mutations
Long-haul (air) travel	Male sex		
Oral contraceptive use	Immobilization		
Certain medications**	Obesity		
	Malignancy		
	IMIDs		
	Adjuvant chemotherapy		

IMIDs = immunomodulatory agents, PNH = paroxysmal nocturnal haemoglobinuria.

* Chronic (inflammatory) diseases, including human immunodeficiency virus (HIV) infection, inflammatory bowel disease, systemic lupus erythematosus, hyperthyroid disease, among others.

** Including tamoxifen, raloxifene, and those containing estrogen.

Recent hospitalization, either for acute medical illness or surgery, accounts for 50% to 60% of the VTE disease burden. Among hospitalized medically ill patients, 75% have multiple risk factors leading to an 8-fold increase in VTE risk when compared to the general population.^[21,22]^ Unprovoked VTE, which occurs due to minor risk factors or in the absence of identifiable risk factors, accounts for 20% to 30% of the disease burden.^[5]^ According to a study at christian medical college Vellore, malignancy was a common risk factor (31%) for DVT followed by postoperative status (30%); among postoperative patients, highest incidence of DVT was noted in patients who underwent general surgery (40.3%) or orthopedic surgery (20.1%). Prolonged intensive care unit stay, vasopressor use, and high Acute Physiology and Chronic Health Evaluation scores were identified as the risk factors.^[23]^

### 1.3. VTE risk assessment

It is necessary to identify individuals who are at increased risk of VTE, either for implementing preventive measures targeted at high-risk groups or for timely initiation of appropriate thromboprophylaxis.^[24,25]^

A risk assessment model (RAM) is a clinical decision-making tool that helps in the identification of individuals at risk of developing VTE with a specific background or clinical condition. Various RAMs have been developed for assessing the risk in medical conditions, such as medical inpatients, major trauma, pregnancy and postpartum, cancer, and lower-extremity cast-immobilization.^[1]^ RAM-based risk stratification and clinical decision-making for patients with VTE facilitates appropriate antithrombotic prophylaxis.^[26]^ Several RAMs such as Caprini, Padua prediction score, Geneva risk score, International Medical Prevention Registry on Venous Thromboembolism (IMPROVE), Khorana, are in use in clinical practice to stratify patients at risk for VTE.^[27–32]^

Although these RAMs have been developed and validated in specific patient populations, the debate about preferred RAM for a specific clinical profile of the patient continues. Thus, a RAM developed for one target population may not apply to another and selection of the precise RAM for a specific population is important.

## 2. Scope of review

Apart from the heterogeneity of the RAMs, there is also a lack of literature identifying the most useful RAM for assessing different patient populations (medically ill, acutely ill, hospitalized, patients undergoing surgery, patients with malignancy). Also, the selection of appropriate RAMs is essential for hospitalized inpatients and outpatients with coronavirus disease 2019 (COVID-19) infection, especially in the current pandemic situation. Further, there is a need to assess the utility of RAMs in the Indian scenario. This review was conducted to: provide an overview of the commonly used RAMs, identify the most suitable RAMs for specific patient populations or risk situations, and assess the utility of and awareness regarding different RAMs in patients at risk for VTE from the global and Indian perspectives.

## 3. Methodology

A literature search was conducted to identify the burden of VTE in India, and globally, along with the currently used VTE-RAMs, using the PubMed database. The literature included in the study was limited to research articles, narrative and systematic reviews, and guidelines published in English. Other research/review papers were identified by reviewing the bibliographies. Literature published in the last 15 years was evaluated and the databases were last accessed in March 2022. This narrative review does not need ethical approval, because no human/patient data was used. The search strings are provided in Appendix S1, Supplemental Digital Content, http://links.lww.com/MD/I177.

## 4. Overview of commonly used RAMs

The use of RAMs to identify high-risk groups and determine the need for prophylaxis is strongly advocated by international guidelines.^[32,33]^ Risk assessment for VTE can be performed either by qualitative or quantitative models.^[34]^

### 4.1. Qualitative/group models for VTE risk assessment

These models assign a group of patients to broad risk categories that are linked to appropriate prophylaxis options for each group. In this model, there is no individualized point-scoring. The most common qualitative model is the “3 bucket” or University of California San Diego Model. Qualitative models are simple to use and implement. They are effective in reducing hospital-associated VTE.^[34]^

According to the 8th edition of the Evidence-Based Clinical Practice Guidelines released by the American College of Chest Physicians (ACCP) about antithrombotic and thrombolytic therapy, patients at risk of VTE can be categorized under the “3 bucket” model (Table 2). The management approach varies accordingly.^[34]^

**Table 2:** Classic “3 bucket” risk category from AT8.^[34]^

Risk category	Patient characteristics
Low risk	Mobile patients undergoing minor surgeries, fully mobile medical patients, and patients under observation with an expected hospital stay <48 hr.
Moderate risk	Patients scheduled to undergo any thoracic, general, urologic or open gynecologic surgery; impaired mobility in medical patients either due to acute illness or other reasons.
High risk	Patients scheduled to undergo surgery for hip fracture, hip or knee arthroplasty, any major spinal surgery, or abdominal-pelvic surgery for cancer; patients with spinal cord injury or multiple major trauma.

AT8 = The eighth edition (AT8) of the American College of Chest Physicians (ACCP) Antithrombotic Therapy and Prevention of Thrombosis Guideline.

### 4.2. Quantitative/individual models for VTE risk assessment

The ninth edition of the Antithrombotic Therapy and Prevention of Thrombosis: ACCP Evidence-Based Clinical Practice Guidelines recommends the use of an individual approach for each patient.^[34,35]^ In this approach, a cumulative point score is calculated based on multiple risk factors. The pioneering RAM in the quantitative category was Caprini, which was extensively used in the 1980s and 1990s for both medical and surgical patients.^[34]^ The Caprini score is a widely used RAM in clinical studies and has been published in 12 languages.^[34,36]^ The underlying reason for the widespread use of the Caprini was its detailed risk assessment at an individual level, which might be considered as more accurate than an assessment based on broad risk factors. Since then, several quantitative RAMs have been developed, such as Padua, Kucher, and IMPROVE.^[34]^ Commonly used individualized RAMs, along with their scoring criteria and corresponding patient populations, are listed in Table 3.

**Table 3:** An overview of a few individualized risk assessment models used in various patient profiles.^[26,27,29,37–53]^

**RAM**	**Patient population**	**Scoring criteria**
Caprini RAM**^[37]^**	Medical, cancer, and surgical patients	Includes 41 risk factors with 1, 2, 3, 4, and 5 scoring points: low risk (0–1), moderate risk (2), high risk (3–4), or highest risk (≥5).
Roger score**^[25]^**	Surgical patients	Considers patient factors, preoperative laboratory values, and operative characteristics. Scores of all factors present are added. Score > 10 represents high risk.
Kucher score**^[27]^**	Medical patients	Includes 8 risk factors with 1, 2, and 3 scoring points: Score ≥ 4 represents “Kucher alert.”
Geneva score**^[26,29,44]^**	Medical patients	Includes 19 risk factors with scoring points of 1 and 2; score 1–2 indicates low risk, score ≥ 3 indicates a high risk.
Risk assessment profile (RAP) score**^[45]^**	Surgical patients (trauma)	Nineteen risk factors under 4 broad categories of underlying conditions, iatrogenic variables, injury-related variables, and age, with 2, 3, and 4 scoring points; score ≥ 5 indicates a high risk.
Padua prediction score**^[37,38]^**	Medical patients	Includes 11 risk factors with 1, 2, and 3 scoring points: low risk (<4), high risk (≥4).
IMPROVE RAM**^[31,46]^**	Acutely ill patients, medical patients	Includes 7 risk factors with 1, 2, and 3 scoring points: Patients are stratified as low risk of VTE (<1%) if their total VTE risk score is <2 points, whereas a score of ≥2 is stratified as high risk.
Khorana score**^[32,33,48–51]^**	All cancer patients, except patients with brain tumors and myelomas	This score stratifies cancer patients into high, intermediate, and low risk of developing VTE during the subsequent 6 months. It includes tumor type, body mass index, pre-chemotherapy hemoglobin, white blood cell, and platelet counts.
COMPASS-CAT RAM**^[52]^**	Cancer outpatients undergoing chemotherapy (for common solid tumor types)	This RAM includes the following variables: (a) time since cancer diagnosis, (b) anthracycline or anti-hormonal therapy, (c) stage of cancer, (d) personal history of VTE, (e) presence of cardiovascular risk factors, (f) recent hospitalization for acute medical illness, (g) central venous catheter, and (h) platelet count.
CONKO score**^[52]^**	Ambulatory solid cancer patients undergoing chemotherapy	The scoring is based on the following parameters: (a) very high-risk tumors (pancreatic or gastric cancer), (b) high risk tumors (lung, bladder, gynecological, lymphoma or testicular), (c) Hb level <10 mg/dL (pre-chemotherapy or use of ESA), (d) pre-chemotherapy WBC count > 11 × 10^9^/L, (e) pre-chemotherapy platelet count ≥ 350 × 10^9^/L, and (f) WHO performance status ≥ 2.
PROTECHT score**^[52]^**	Ambulatory solid cancer patients undergoing chemotherapy	The scoring is based on the following parameters: (a) very high-risk tumors (pancreatic or gastric cancer), (b) high risk tumors (lung, bladder, gynecological, lymphoma or testicular), (c) Hb level < 10 mg/dL (pre-chemotherapy or use of ESA), (d) pre-chemotherapy WBC count > 11 × 10^9^/L, (e) pre-chemotherapy platelet count ≥ 350 × 10^9^/L, (f) BMI > 35 kg/m^2^, (g) gemcitabine chemotherapy, and (h) platinum-based chemotherapy.
RCOG score**^[53]^**	Obstetric population	Categorizes pregnant or postpartum women into high, intermediate and low risk categories based on defined set of risk factors. For pregnant women: high risk if previous VTE event, except the one related to major surgery; intermediate risk if hospital admission, previous single VTE event related to major surgery, high-risk thrombophilia with no VTE, presence of medical comorbidities, any surgical procedure, or OHSS; and low risk for presence of ≥4 of the following risk factors: age > 35 yr, smoker, gross varicose veins, parity ≥ 3, current pre-eclampsia, immobility, low-risk thrombophilia, family history of VTE in first degree relative, multiple pregnancy, and IVF/ART.

ART = assisted reproductive technology, BMI = body mass index, COMPASS-CAT = comparison of methods for thromboembolic risk assessment with clinical perceptions and AWARENESS in real life patients-cancer associated thrombosis, ESA = erythropoietin stimulating agents, Hb = hemoglobin, IMPROVE = International Medical Prevention Registry on Venous Thromboembolism, IVF = in vitro fertilization, OHSS = ovarian hyperstimulation syndrome, RAM = risk assessment model, RCOG = Royal College of Obstetricians and Gynecologists, VTE = venous thromboembolism, WBC = white blood cell, WHO = World Health Organization.

## 5. RAMs according to underlying condition

An ideal RAM is the one that has been externally validated through clinical studies for the identification of at-risk patients, improves the rates of clinical events and prophylaxis, and is cost-effective. Identifying a patient at risk for a VTE is the prerequisite before initiation of a thromboprophylaxis. Hence implementation of the RAM is quintessential to reduce VTE incidence.^[26]^

In the current review, commonly used RAMs have been categorized under 5 groups of VTE risk conditions/populations: patients with medical illness, surgical condition, cancer, pregnant women, and COVID-19 infection (Tables 4 and 5).

**Table 4:** Studies evaluating commonly used risk assessment models for COVID-19 patients.^[72,75,76]^

**RAM used**	**Studies evaluating RAM**	**Study design**	**Study outcomes**
COVID-19 patients			
Padua score	Zeng et al, 2020^[75]^	A prospective single-center study involving 274 COVID-19 patients	Patients with higher Padua scores had a significant survival disadvantage. Critical patients showed a higher Padua score (6 vs 2, *P* < .001) versus severe patients.
	Xu et al, 2020^[76]^	A single-center retrospective observational study involving 138 COVID-19 patients	The incidence of VTE among critically ill patients was around 20%. According to the Padua score, 16.67% of patients with COVID-19 were at high risk for VTE.
Caprini score	Tsaplin et al, 2020^[72]^	A single-center retrospective analysis of 168 COVID-19 patients	VTE was diagnosed in 6.5% of patients. The study identified a significant correlation between the Caprini score and the risk of VTE or unfavorable outcomes in COVID-19 patients.

COVID-19 = coronavirus disease 2019, RAM = risk assessment model, VTE = venous thromboembolism.

**Table 5:** Risk assessment models validated in India.^[77–81]^

**RAM used**	**Studies evaluating RAM**	**Study design**	**Study outcomes**
Caprini **score**	Chandrakumar et al, 2016^[79]^	A single-center prospective study of 1-year duration in Kerala, South India. The study enrolled 400 patients admitted for surgery.	As per the Caprini score, the number of patients with low, moderate, and high risk were 24%, 35%, and 41%, respectively.
	Panda et al, 2017^[80]^	A single-center prospective study, of one-year duration in Maharashtra, India. The study enrolled 210 patients admitted to the ICU and surgery ward.	21.3%, 33.3%, and 45.3% of critically ill patients were classified as having moderate, higher, and highest VTE risks. 13.3%, 36.6%, and 50% of postsurgical patients were categorized as moderate, higher, and highest VTE risk.
Adapted Caprini **score**	Bilgi et al, 2016^[77]^	A single-center prospective observational study of 1-year duration in Pondicherry, South India. The study enrolled 301 surgical patients.	The risk of VTE was significantly higher among the >8 score group as compared to 3–4 (*P* < .001), 5–6 (*P* < .001), or 7–8 (*P* = .002) score groups. As compared to 3–4 or 5–6 score groups, patients with 7–8 scores were more likely to develop VTE.
Padua **score**	Ali et al, 2018^[78]^	A single-center retrospective observational study including 100 medical inpatients	69% of patients were at high risk of developing VTE and 31% were at low risk.
Modified Padua **score**	Hussaini et al, 2019^[81]^	A single-center prospective observational study conducted over 6 months, involving 100 clinical and surgical patients at risk of VTE	41% of patients were identified to be at high risk, 18% at moderate risk, and 41% at low risk of VTE.

ICU = intensive care unit, RAM = risk assessment model, VTE = venous thromboembolism.

### 5.1. Medical patients

Up to 50% of all VTE cases that occur each year are noted during hospitalization,^[54]^ whereas 75% of fatal VTE occurs in medically ill, hospitalized patients.^[55]^ Although the period of VTE risk may extend up to 90 days after discharge, the majority (80%) of VTE occur within the first 45 days after hospital discharge.^[56]^ Medically ill patients have increased VTE-related readmission rates, which reach up to 28% 6 months after hospital admission.^[55]^

Several RAMs have been studied for appropriate utilization of thromboprophylaxis modalities in medically ill patients. The American Society of Hematology 2018 guideline recommends the 2 most studied models namely Padua prediction score and IMPROVE VTE RAM, both of which are externally validated and showed fair discrimination in identifying medical inpatients who are and are not at increased risk for VTE.^[55]^ Furthermore the investigators of IMPROVE have also developed an externally validated bleeding risk RAM which can identify the acutely ill patients who are at an increased risk of bleeding. The Padua model helps in differentiating medical patients who are at a high risk of VTE from those at low risk of VTE. In addition, this RAM improves the stratification of thromboembolic risk in hospitalized medical patients, as compared to the other validated scores.^[30,55]^

According to a single-center study that included 2282 hospitalized patients in the Rheumatology Department, 188 and 2094 patients were categorized as high-risk and low-risk based on Padua score, respectively. The optimal sensitivity and specificity of Padua score were 60% and 82.5%, respectively.^[57]^

IMPROVE VTE RAM was obtained from a large international registry of 15,156 acutely ill, hospitalized medical patients. Large-scale external validation studies have demonstrated good calibration and discrimination with IMPROVE RAM, suggesting that the IMPROVE associative VTE RAM is reliable and stratifies VTE risk.^[55,56]^

Padua score has been advocated by the ACCP guidelines for stratifying non-surgical patients. Other RAMs such as IMPROVE have also been suggested for preventing VTE in hospitalized medical patients. It has also been reported that Padua score can detect a variation in VTE risk of approximately 30-fold among acutely ill medical inpatients.^[58,59]^

### 5.2. Surgical patients

According to a recent study, the overall risk of VTE in patients who underwent surgery was 4.6% and ranged between 2.3% and 9.3% based on the type of surgery. The highest risk of recurrence was reported in major orthopedic, cancer-related, and heart-lung procedures and gastrointestinal surgeries.^[60]^ The Caprini Risk Score is a comprehensive list of 41 thrombosis risk factors validated in >5 million patients.^[61]^ It is the most widely used and validated RAM for surgical patients.^[40,41,43]^ A retrospective study on arthroplasty patients reported that Caprini RAM provides an effective, consistent, and accurate method of VTE risk stratification in these surgical patients. In this retrospective study, Karuss et al reported a specificity of 0.64 (95% confidence interval [CI], 0.61–0.67) and sensitivity of 0.88 (exact 95% CI: 0.47–1.00) for the Caprini model.^[41]^ In a case-controlled review of perioperative patients involving 18 VTE cases and 171 matched controls, the sensitivity of the Caprini and Padua RAMs were reported to be 88.9% and 61.1%, respectively.^[40,62]^ The 2012 ACCP guidelines recommends VTE prevention in non-orthopedic surgical patients, by Caprini score and Rogers score.^[63]^

### 5.3. Cancer patients

VTE in patients with cancer may notably impact mortality and morbidity. Moreover, VTE is the second leading cause of death in these patients.^[64]^ Patients with cancer have a 4- to 7-fold increased risk of VTE compared to patients without cancer. Approximately 20% to 30% of all VTE cases occur in patients with cancer.^[65]^ Assessing the thrombotic burden in patients with active cancer, referring to those who have been diagnosed with a current or recent malignancy, those with metastatic disease, or those receiving anticancer treatment, remains a challenge, as patients with active cancer may experience thromboembolic and bleeding complications.^[66]^ An individualized assessment of every patient’s profile is therefore required.^[67]^ Risk stratification tools have been developed to identify patients with cancer who are at a high risk of requiring thromboprophylaxis. The best-validated tool is a score proposed by Khorana and colleagues. The Khorana score is the first and the most widely used score that identifies ambulatory cancer patients at increased risk of VTE during chemotherapy.^[68]^ The American Society of Clinical Oncology 2019 guidelines recommend the Khorana RAM score for ambulatory patients with solid tumors on treatment with systemic therapy.^[69]^ The PROTECHT score is a modified Khorana risk assessment score, which has been designed by adding platinum-based or gemcitabine-based chemotherapy to the predictive variables of the Khorana score.^[70]^ The CONKO score is another risk score developed by adding the World Health Organization performance status (+1 point for the World Health Organization performance status of ≥2) to the Khorana score.^[71]^ The COMPASS-CAT score provides an accurate RAM for VTE in outpatients on anticancer therapy and allows stratification of patients at high and low/intermediate risk for VTE. It includes reliable and easily collected VTE predictors associated with cancer evolution and its treatments and with patient characteristics and comorbidities. It is valid for the most frequently occurring types of solid tumors, which greatly impact the VTE burden. This RAM can be used while the patient is on chemotherapy, thus permitting reevaluation of VTE risk during the patient’s course of disease. The COMPASS-CAT RAM can easily recognize patients with cancer on anticancer treatment at low or intermediate risk of VTE and rule out the need for an antithrombotic primary prevention strategy.^[72]^

### 5.4. COVID-19 infection

The current pandemic of highly infectious COVID-19 is caused by the severe acute respiratory syndrome coronavirus 2. In case of severe infection, it leads to the development of acute respiratory distress syndrome or pneumonia.^[73]^

A recent meta-analysis has reported that the 21% of patients with COVID-19 developed VTE.^[74]^ In a study by Lee AD et al, the incidence of VTE in India (pre-COVID era) was found to be 17.46 per 10,000 hospital admissions.^[13]^ A systematic review and meta-analysis (with a total of 41,768 patients) was conducted to compare the incidence of VTE in COVID-19 cohorts with that of non-COVID-19 cohorts. COVID-19 and non-COVID-19 cohorts did not differ significantly in VTE risk, except in the hospitalized intensive care unit subgroups.^[75]^ A higher mortality rate was observed in COVID-19 patients with VTE than in patients without VTE (23% [95% CI: 14–32%] vs 13% [95% CI: 6–22%]), and a meta-analysis revealed that the thromboembolism-related mortality risk increased by 74% in patients with COVID-19 infection.^[74]^

The inflammatory process, lung injury, and cytokine storm associated with COVID-19 increase the VTE risk.^[76]^ Owing to the high risk of VTE, a few RAMs have been validated in COVID-19 patients, such as the Padua and Caprini scores (Table 4).^[73,76,77]^ The Caprini score was found to be significantly associated with VTE (*P* < .001) and a higher Caprini score was associated with unfavorable outcomes in these patients.^[73]^ A higher Padua score was also associated with poor prognosis and survival disadvantage in hospitalized COVID-19 patients.^[76]^ However, the application of RAMs for patients with COVID-19 is still in a nascent state globally.

## 6. Utility of RAMs in India

Earlier studies have reported a higher incidence of VTE in Western countries compared to Asian countries, including India.^[13]^ However, recent studies have shown that VTE in India is not as infrequent and uncommon as perceived previously.^[78]^ Thromboprophylaxis is the most important strategy to improve patient safety in hospitals and prevent hospital-related deaths due to VTE. However, evidence indicates that there is a major underutilization of prophylaxis in India as compared to global rates.^[79]^ Currently, VTE risk assessment in India is inadequately utilized. Even though treating physicians are aware of the importance of RAMs for VTE risk assessment, RAMs remain underutilized in routine clinical practice.^[13]^ This reiterates the need for increasing awareness about VTE risk, identifying risk factors, using RAMs, and improving the effective implementation of appropriate thromboprophylaxis in patients at risk of VTE. This will ensure the successful management of VTE and prevent VTE-associated morbidity and mortality.^[79]^ A few studies have been performed in India on VTE risk assessment (Table 5).

The studies performed in India are limited and include mostly surgical patients. There is a lack of studies on medical patients for VTE incidence and risk assessment.^[82]^ Mostly, the Caprini RAM has been utilized in the reported studies. Although RAMs have been quite extensively validated in the Western population, they have not been validated in the Indian context. As there are differences in race, lifestyle, and genetic make-up, the risk factors in the Caucasians and the Indian population may not be the same. Furthermore, India is a diverse country. Hence, large multicenter studies are required to analyze clinical data on patients, screen VTE risk factors, determine their degree of influence, formulate corresponding risk levels, and determine appropriate prevention methods. Moreover, studies on special risk populations, such as cancer patients or pregnant women, are lacking in India. In India, VTE risk assessment also needs to include a systematic approach to evidence-based prophylaxis in patients with COVID-19 infection.

The commonly used VTE-RAMs have been developed based on the risk factors of the Caucasians. Although the acquired risk factors in Asians are similar to the Western population, the heritable or genetic risk factors are different. For example, the prevalence of heritable risk factors, such as antithrombin III deficiencies, and prevalence of protein S and protein C deficiencies are higher in Asian population, prothrombin G20210A polymorphisms, and factor V Leiden are specific risk factors for Caucasians.^[7]^ Owing to such variations in risk factors, the common VTE-RAMs fall short of accurate identification of at-risk VTE patients in the Asian population. This limitation of existing VTE-RAMs in the context of the Asian population warrants further studies in this population.^[7]^ Moreover, since the acquired risk factors are similar across the Asian and Western populations, RAMs, such as Caprini and Padua, can be used for the Asian population. To optimize the identification of at-risk patients and thromboprophylaxis, individualization of VTE risk assessment has been emphasized by the Asian guidelines.^[83]^ A similar approach would be beneficial for the implementation of VTE RAMs in the Indian context.

Various studies from India have emphasized the need for improved awareness of VTE and its risk assessment in the country, both for the patients and physicians.^[17,79,84,85]^ According to a recent study, the use of thromboprophylaxis in DVT patients was not based on any risk assessment score.^[85]^ Possibly, the use of RAM for VTE risk assessment and thromboprophylaxis is not perceived as important in the overall Indian medical community, or the implementation of RAM is perceived to be tedious. This is fueled by low awareness regarding VTE symptoms in patients,^[81]^ and the fear of risks involved with thromboprophylaxis, such as the increased risk of bleeding.^[83]^ Therefore, efforts are needed to increase VTE awareness through multidisciplinary educational programs.^[81]^

Regarding the prophylaxis of VTE patients, prolonged anticoagulation therapy is not recommended in patients who are at high risk of bleeding. Therefore, for determining the appropriate duration of anticoagulation therapy in VTE patients, it is important to identify high-risk patients for bleeding. Many healthcare providers are unaware of the bleeding risk scores for estimating the bleeding risk when anticoagulation therapy is initiated. IMPROVE BLEED and HAS BLED (Hypertension, Abnormal Renal/Liver Function, Stroke, Bleeding History or Predisposition, Labile INR, Elderly, Drugs/Alcohol Concomitantly) are a few scores that can identify patients at a high risk of bleeding and further guide healthcare providers on the choosing of a pharmacological or mechanical thromboprophylaxis. There is no well-validated RAM in this context, and the clinical utilization of any bleeding risk scores in Indian scenario is yet to be realized.

In summary, although several validated RAMs have been developed globally for different patient populations and conditions, to facilitate early diagnosis and prophylaxis for VTE, systematic efforts to identify patients at risk of VTE by utilizing appropriate RAMs in India needs to be strengthened.^[12]^ Further, since some of the risk factors for VTE predisposition are markedly different in the Indian population, as compared to the Western counterparts, there is also an unmet need of large-scale multicenter validation studies for existing RAMs.^[13,15,17,18]^ Nevertheless, routine implementation of available RAMs for VTE risk assessments in different patient populations and their appropriate use in the Indian population is the need of the hour. Furthermore, developing a RAM specifically for the Indian population would also be of paramount importance.

## 7. Conclusion

RAMs are essential and dynamic tools for risk-stratification, prevention, and overall management of VTE. The risk of VTE should be assessed not only at the time of hospitalization, but also at discharge. From existing reports, it is evident that the utilization of RAMs in Indian clinical practice is limited. It is also observed that the majority of global RAM validation is performed in retrospective studies. Therefore, large-sample prospective and multicentric studies to ascertain the effectiveness of each RAM is essential. Also, as an ideal RAM considers cost-effectiveness of care, future studies evaluating the effect of RAMs on financial burden are warranted.

## Acknowledgments

We would like to acknowledge BioQuest Solutions Pvt. Ltd., Bangalore, for providing medical writing and editorial support in the preparation of this manuscript paid for by Sanofi India.

## Author contributions

Y Mehta and A Bhave have contributed to the conceptualization and narrative of the review. Both authors have contributed to interpretation of data; have read and reviewed the final draft of this manuscript; take responsibility for the integrity and accuracy of this manuscript; and have given their approval for this version to be published.

## Supplementary Material


