# Supplementary material for: A review of venous thromboembolism risk assessment models for different patient populations: What we know and don’t!
Source: Medicine (Baltimore). 2023 Jan 13;102(2):e32398. doi: 10.1097/MD.0000000000032398 (PMC9839272; doi:10.1097/MD.0000000000032398)
Supplement: Supplementary file 1 [file medi-102-e32398-s001.pdf]

Supplemental Digital Content (Appendix 1): Search strategy

No.	Search strategies
1	("Venous thromboembolism" OR Thromboembolism OR Thrombosis OR VTE) AND ("Risk assessment model" OR RAM OR "Risk assessment tool" OR "VTE assessment tool")
2	("Venous thromboembolism" OR Thromboembolism OR Thrombosis OR VTE OR "Deep-vein thrombosis" OR "Pulmonary embolism") AND ("Risk assessment model" OR RAM OR "Risk assessment tool")
3	("Venous thromboembolism" OR Thromboembolism OR Thrombosis OR VTE OR "Deep-vein thrombosis" OR "Pulmonary embolism") AND ("Risk assessment model" or RAM OR "Risk assessment tool" OR "VTE assessment tool" OR "Risk analysis model")
4	("Venous thromboembolism" OR Thromboembolism OR Thrombosis OR VTE OR "Deep-vein thrombosis" OR "Pulmonary embolism") AND ("Risk assessment model" OR RAM OR "Risk assessment tool" OR "VTE assessment tool" OR "Risk analysis model") AND Caprini OR Padua OR Kucher OR "Chao–Yang" OR Khorana OR "4-Element" OR "IMPROVE-RAM" OR "Full logistic" OR Geneva OR "Multivariable model" OR Intermountain OR TESS OR “DoH RAM”)
5	("Venous thromboembolism" OR Thromboembolism OR Thrombosis OR VTE OR "Deep-vein thrombosis" OR "Pulmonary embolism") AND ("Risk assessment model" OR RAM OR "Risk assessment tool" OR "VTE assessment tool" OR "Risk analysis model") AND India
6	("Venous thromboembolism" OR Thromboembolism OR Thrombosis OR VTE OR "Deep-vein thrombosis" OR "Pulmonary embolism") AND ("Risk assessment model" OR RAM OR "Risk assessment tool" OR "VTE assessment tool" OR "Risk analysis model") AND (“COVID-19” OR “Corona Virus Disease” OR “COVID”)
